# Proteoglycans Act as Cellular Hepatitis Delta Virus Attachment Receptors

**DOI:** 10.1371/journal.pone.0058340

**Published:** 2013-03-07

**Authors:** Oscar Lamas Longarela, Tobias T. Schmidt, Katrin Schöneweis, Raffaella Romeo, Heiner Wedemeyer, Stephan Urban, Andreas Schulze

**Affiliations:** 1 Department of Infectious Diseases, Molecular Virology, University Hospital Heidelberg, Heidelberg, Baden-Württemberg, Germany; 2 First Division of Gastroenterology, Fondazione IRCCS Ca’Granda Ospedale Maggiore Policlinico, University of Milan, Milan, Italy; 3 Department of Gastroenterology, Hepatology and Endocrinology, Hannover Medical School, Hannover, Germany; Inserm, U1052, UMR 5286, France

## Abstract

The hepatitis delta virus (HDV) is a small, defective RNA virus that requires the presence of the hepatitis B virus (HBV) for its life cycle. Worldwide more than 15 million people are co-infected with HBV and HDV. Although much effort has been made, the early steps of the HBV/HDV entry process, including hepatocyte attachment and receptor interaction are still not fully understood. Numerous possible cellular HBV/HDV binding partners have been described over the last years; however, so far only heparan sulfate proteoglycans have been functionally confirmed as cell-associated HBV attachment factors. Recently, it has been suggested that ionotrophic purinergic receptors (P2XR) participate as receptors in HBV/HDV entry. Using the HBV/HDV susceptible HepaRG cell line and primary human hepatocytes (PHH), we here demonstrate that HDV entry into hepatocytes depends on the interaction with the glycosaminoglycan (GAG) side chains of cellular heparan sulfate proteoglycans. We furthermore provide evidence that P2XR are not involved in HBV/HDV entry and that effects observed with inhibitors for these receptors are a consequence of their negative charge. HDV infection was abrogated by soluble GAGs and other highly sulfated compounds. Enzymatic removal of defined carbohydrate structures from the cell surface using heparinase III or the obstruction of GAG synthesis by sodium chlorate inhibited HDV infection of HepaRG cells. Highly sulfated P2XR antagonists blocked HBV/HDV infection of HepaRG cells and PHH. In contrast, no effect on HBV/HDV infection was found when uncharged P2XR antagonists or agonists were applied. In summary, HDV infection, comparable to HBV infection, requires binding to the carbohydrate side chains of hepatocyte-associated heparan sulfate proteoglycans as attachment receptors, while P2XR are not actively involved.

## Introduction

The hepatitis delta virus (HDV) is a small, defective RNA virus. It can propagate only in presence of the hepatitis B virus (HBV) either by simultaneous transmission of both viruses or by superinfection of an established HBV carrier as HDV is dependent on the presence of the HBV envelope proteins for assembly and spread [1], [2]. HDV is composed of an envelope containing the three HBV proteins named large (L), middle (M) and small (S) surrounding a nucleocapsid, consisting of the single-stranded, circular RNA and the hepatitis D antigen (HDAg) [3]. Due to the usage of the HBV envelope proteins, it is thought that HBV and HDV share the same cellular receptor molecule(s). Several studies demonstrated that a major HBV/HDV infectivity determinant is located in the N-terminal part of the preS1-domain of the HBV L-protein [4]–[6]. The most compelling are studies using reverse genetics showing that the integrity of amino acids (aa) 2 to 75 is essential for infectivity [4], [7], [8]. Acylated peptides encompassing the N-terminal 47 aa of the preS1-domain block HBV and HDV infection *in vitro* and *in vivo* [4], [5], [9]–[11]. Fine-mapping of the sequence requirements showed that N-terminal myristoylation and aa 9–15 are necessary for receptor interaction [12], [13]. A second infectivity element is located in the antigenic loop (AGL) of the S domain of the HBV envelope proteins [14], [15]. The study by Le Duff *et al.* suggests that the determinants in the preS1-region and the AGL act independently on HBV/HDV entry [16].

In the last years, a number of cellular proteins have been suggested as HBV receptor molecule(s) [17]. Recently, sodium taurocholate cotransporting polypeptide (NTCP) was identified as functional, specific receptor for HBV and HDV [18]. Furthermore, we and others previously showed that the glycosaminoglycan (GAG) side chains of heparan sulfate proteoglycans (HSPG) act as attachment receptor for HBV on the surface of hepatocytes [19], [20]. GAGs are unbranched polysaccharides composed of hexosamine/hexuronic acid repeats that acquire negative charges through N- and O-sulfation. They are bound to core proteins or lipids to form glycoconjugates, e.g. proteoglycans. GAGs are ubiquitously expressed but display a cell type–specific structural heterogeneity, reflected in variations in length and composition of the carbohydrate chains that determine the strength and specificity of the contacts [21]–[24].

Recently, another possible HBV/HDV binding partner has been suggested, the purinergic receptors (P2XR) [25]. P2XR are ATP-gated membrane ion channels with multiple functions including afferent sensation, autocrine feedback and inflammation [26]–[28]. They are expressed in a wide range of tissues. The P2X_4_ and P2X_7_ isoforms have been identified in primary rat hepatocytes and the human hepatoma cell line Huh7 [29]. In their study, Taylor *et al.* used five P2XR antagonists to investigate whether P2XR are involved in HBV/HDV entry.

Here, we examined the role of cellular proteoglycans in the HDV entry process, by using a set of charged and non-charged compounds to interfere with HDV infection of HepaRG cells and primary human hepatocytes (PHH). At the same time, we tested the role of P2XR on HBV and HDV infection in more detail by applying charged and uncharged antagonists and agonists. We provide evidence that HDV infection depends on the initial interaction with GAG side chains of cell surface associated HSPG as attachment receptors. We furthermore propose that the observed effect of negatively-charged P2XR antagonists is mediated by their charge (by inhibiting interaction with cellular proteoglycans) and not by a role of P2XR in the HBV/HDV entry process.

## Materials and Methods

### Reagents

The HBV preS1-peptide preS/2-48^myr^ was synthesized and purified as previously described [13]. Chondroitin sulfate A and C, dextran, dextran sulfate, heparin, ivermectin, N-acetyl-de-O-sulfated heparin, poly-L-lysine, pyridoxal-phosphate-6-azophenyl-2′,4′-disulfonate (PPADS), 2,4-thiazolidinedione (AZ11645373), sodium chlorate, suramin and heparinase III were purchased from Sigma-Aldrich (Germany). Pentosan polysulfate was bought from beneArzneimittel GmbH (Germany).

### Cell Lines

PHH were provided by Thomas Weiss (Regensburg, Germany). Tissue samples from liver resections were obtained from patients undergoing partial hepatectomy. Experimental procedures were performed according to Human Tissue and Cell Research Foundation guidelines, with written informed patient consent approved by the Ethical Committee of the University of Regensburg (Regensburg, Germany). Upon arrival, PHH were washed in Williams’ medium E supplemented with 10% heat-inactivated fetal calf serum (FCS), 50 U of penicillin/ml, 50 µg of streptomycin/ml, 5 µg of insulin/ml, and 50 µM hydrocortisone hemisuccinate and 1.5% dimethyl sulfoxide (DMSO). After 24 h cultivation, the cells were washed with phosphate buffered saline (PBS) and fresh media without FCS was added. HepaRG cells were grown in Williams’ medium E supplemented with 10% heat-inactivated FCS, 50 U of penicillin/ml, 50 µg of streptomycin/ml, 5 µg of insulin/ml, and 50 µM hydrocortisone hemisuccinate. The cells were split every 2 weeks at a ratio of 1∶5. Fourteen days before infection, cell differentiation was induced by adding 1.5% DMSO to the maintenance medium [30]. The medium was exchanged every 3 days.

### HDV and HBV Infection of HepaRG Cells and PHH

As inoculum for HDV infections, sera from HDV carriers (GTA7, BGJN, APIS21c and MPA4) were used. Approximately 100-fold concentrated HepAD38 cell culture supernatants served as stocks for HBV infections [31]. The HBV inoculum was prepared by precipitating viral particles from cell culture supernatants with 6% polyethylene glycol 8000 (Sigma-Aldrich) for 12 to 16 h at 4°C. The precipitates were recovered by centrifugation at 7,000×*g* for 1 h at 4°C and resuspended in PBS supplemented with 25% FCS. Aliquots were stored at -80°C. For HBV and HDV infection, differentiated HepaRG cells (1×10^6^ cells/well of a 12-well plate) or PHH (8×10^5^ cells/well of a 12-well plate) were incubated with 9×10^4^ HDV-RNA copies (as quantified by qRT-PCR at Hannover Medical Centre) or a 20-fold dilution of the concentrated HBV stock in medium (corresponding to ≈ 4×10^10^ genome equivalents) in presence of 4% polyethylene glycol 8000 for 16 h at 37°C. At the end of the incubation, the cells were washed extensively and further cultivated. Medium was exchanged every 3 days. For infection competition experiments, HepaRG cells or PHH were incubated for 30 min at 37°C with the HBV preS1-peptide preS/2-48^myr^ or GAGs and for 1 h at 37°C with the HBV preS1-peptide preS/2-48^myr^ or the P2XR antagonists/agonists; followed by incubation of the cells with HBV or HDV for 16 h at 37°C in presence of the compounds. For cell pre-incubation studies, HepaRG cells were incubated for 30 min at 37°C with the respective substance followed by washing of the cells and virus inoculation in absence of the compounds for 8 h at 37°C. For virus pre-incubation experiments, HDV was incubated for 1 h at 37°C with GAGs. Unbound GAGs were removed by ultrafiltration. Infection was performed with the pretreated HDV particles. Virus inoculation with HepaRG cells was shortened to 8 h at 37°C. For post-incubation studies, HepaRG cells were washed after virus inoculation and the compounds were added. Incubation with the substances was performed for 24 h at 37°C. To quantify the HBV infection, hepatitis B surface antigen (HBsAg) secreted into the culture supernatant from day 8 to 11 post infection (p.i.) was quantified by enzyme-linked immunosorbent assay ([ELISA] AxSYM; Abbott). Hepatitis B e antigen (HBeAg) was determined by the ADVIA Centaur XP™ automated chemiluminescence system (Siemens). HDV infection was quantified by HDV-specific immunofluorescence. All competition experiments were performed in duplicate and repeated at least two times independently.

### Inhibition of GAG Sulfation and Enzymatic Removal of GAG Chains

To prevent the sulfation of GAG side chains of cell-associated proteoglycans, HepaRG cells were cultivated for 48 h at 37°C in presence of sodium chlorate. Subsequent virus inoculation was performed in presence of the inhibitor. Enzymatic removal of GAG structures was achieved by heparinase III treatment of HepaRG cells. The lyase was solved according to the manufacturers’ protocol, added to prewashed cells, and incubated for 1 h at 37°C. Subsequently, the cells were washed and infection was performed in presence of the enzyme. Enzyme concentrations are expressed in international units (IU) per milliliter (1 IU ≈ 600 Sigma units).

### HDV-specific Immunofluorescence (IF) Analyses

6 days p.i., PHH or HepaRG cells were washed, fixed with 4% paraformaldehyde in PBS for 30 min at room temperature, and permeabilized with 0.25% (vol/vol) Triton X-100 in PBS for 30 min at room temperature. A serum from an HDV carrier (GEAO) containing HDV-specific antibodies was added at a dilution of 1∶3,000 in PBS containing 5% skim milk powder. Following overnight incubation at 4°C, cells were washed and incubated in the dark for 1 h at room temperature with a 1∶1,000 dilution of a goat-anti-human Alexa Fluor 555-conjugated secondary antibody (Invitrogen/Molecular Probes) in PBS containing 5% skim milk powder. After washing, cells were incubated for 5 min with a 1 µg/ml solution of 4′6′-diamidino-2′-phenylindole dihydrochloride (DAPI; Roche Applied Science). Cells were washed with PBS. Images were acquired with an inverted fluorescence microscope (Leica, Germany). All images were made under the same settings and afterwards handled identically. The images were analyzed using the ImageJ software. The nucleus counter function of the Wright Cell Imaging Facility (WCIF) plug-in was used to count the number of nuclei. The cell counter plug-in was used to count the number of HDAg-positive cells. For each compound and concentration, four to forty image sets were analyzed.

### HDV-specific Western Blot Analyses

5 days p.i. HepaRG cells were washed with PBS and lysed with a buffer containing 25 mM Tris-HCl (pH 7.4), 250 mM NaCl, 5 mM EDTA and 1% Nonidet P-40. Proteins were subjected to SDS-PAGE under reducing conditions. For Western Blot analysis, GEAO serum (dilution 1∶3,000 in TBST) and IR Dye 800–conjugated goat anti-human IgG (Rockland; dilution 1∶20,000 in TBST) were used as primary and secondary antibody. Visualization was performed by an Odyssey infrared imaging system (LI-COR).

## Results

### Highly Negatively Charged GAGs Abolish the in vitro HDV Infection

**Figure 1:**
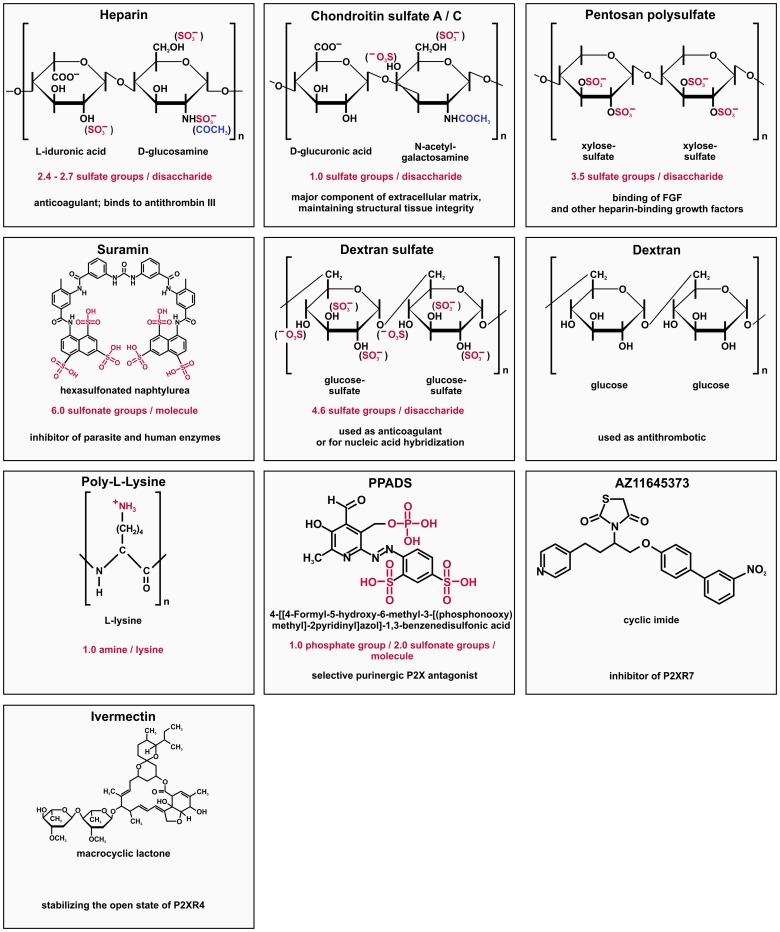
Chemical structures, charge and function of the compounds used in the study. Listed are the structures of the repeating units (disaccharide or lysine) or the individual molecules. Charged groups are depicted in red. Possible acetylations are marked in blue. The number of charged groups per repeating unit or molecule and the function of the substance are listed. Not included are derivatives of the compounds.

**Figure 2:**
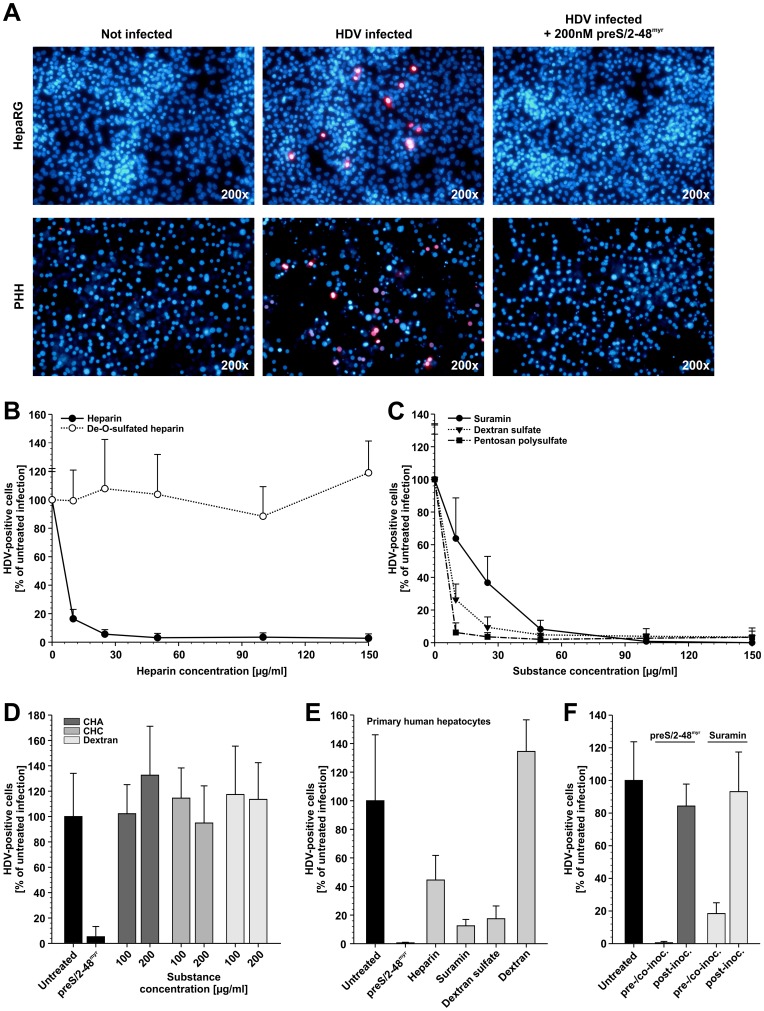
Highly-sulfated GAGs interfere with the in vitro HDV infection. A ) HepaRG cells or PHH were infected with HDV for 16 h at 37°C. Uninfected cells or cells treated with 200 nM preS/2-48^myr^ peptide during virus inoculation were used as controls. Overlays of HDV-specific IF (red) and nucleus staining (blue) for the respective infections on HepaRG cells (top) or PHH (bottom) on day 5 p.i. are depicted. **B–E**) HepaRG cells (B–D) or PHH (E) were pre-incubated for 30 min at 37°C with the indicated substances followed by HDV infection for 16 h at 37°C in presence of the compounds. HDV-specific IF was performed on day 6 p.i. Nuclei were stained with DAPI. The number of HDAg-positive cells and the total cell number were determined. The results are depicted as percentage of infected cells in comparison to the untreated control. **F**) HepaRG cells were pre-incubated for 30 min at 37°C with 500 nM preS/2-48^myr^ or 100 µg/ml suramin. HDV inoculation was performed for 16 h at 37°C in presence of the substances (pre−/co-inoc.). In parallel HepaRG cells were infected with HDV for 16 h at 37°C without treatment. After inoculation, the cells were washed and 500 nM preS/2-48^myr^ or 100 µg/ml suramin was added to the untreated, HDV infected cells. After 24 h at 37°C all cells were washed and further cultivated. HDV-specific IF was performed on day 6 p.i. Nuclei were stained with DAPI. The number of HDAg-positive cells and the total cell number were determined. The results are depicted as percentage of infected cell in comparison to the untreated control. In total, 735258 cells were counted for the experiments.

To test whether soluble GAGs interfere with HDV infection, we performed competition experiments on HepaRG cells and PHH using HDV-containing patient sera as infectious inoculum and increasing concentrations of GAGs and other charged compounds. Detailed information about the compounds used in this study can be found in figure 1. HDV-specific IF of HepaRG cells (upper row) or PHH (lower row) on day 5 post HDV infection is shown in figure 2A. Approximately 3% of HepaRG cells and 13% of PHH became infected under the chosen conditions. HDV infection could be blocked using the previously characterized HBV preS1-derived lipopeptide preS/2-48^myr^ [11] excluding unspecific entry.

Heparin, the prototype member of the GAG family, abolished the HDV infection of HepaRG cells in a dose-dependent manner with a concentration that inhibits 50% (IC_50_) of approximately 6 µg/ml (figure 2B). Removal of the O-sulfation of heparin led to a complete abrogation of the inhibitory activity, indicating that the degree of negative charge is important. To further address this point and investigate the contribution of the carbohydrate backbone, different highly sulfated compounds (suramin, dextran sulfate and pentosan polysulfate) (figure 2C) as well as low (chondroitin sulfate A and C), respectively non-charged (dextran) substances (figure 2D) were tested. Suramin, dextran sulfate and pentosan polysulfate abolished the HDV infection of HepaRG cells with IC_50_ values of ∼ 18 µg/ml, ∼ 7 µg/ml and ∼ 5 µg/ml, respectively. However, neither chondroitin sulfate A and C, nor dextran had an influence on the HDV infection at concentrations up to 200 µg/ml, substantiating the necessity of a high degree of sulfation for complete inhibition. These observations were confirmed in PHH using 100 µg/ml heparin, suramin, dextran sulfate and dextran (figure 2E). While heparin, suramin and dextran sulfate decreased the HDV infection by 55 to 90%, dextran was inactive. Suramin has been described to block DHBV and HDV infection, having only a slight effect on the HDV RNA synthesis once HDV infection is established [32], [33]. We confirmed these results by adding suramin (100 µg/ml) for 24 h to HepaRG cells immediately after HDV inoculation (figure 2F). Like suramin, preS/2-48^myr^ had no effect when given after virus inoculation. This emphasizes the role of suramin in an early step of the HDV infection. The IC_50_ values obtained for HDV infection are in the same range as those previously obtained by our group for HBV infection [20].

### Neutralization of Negative Charges on HepaRG Cells Abrogates HDV Infection

**Figure 3:**
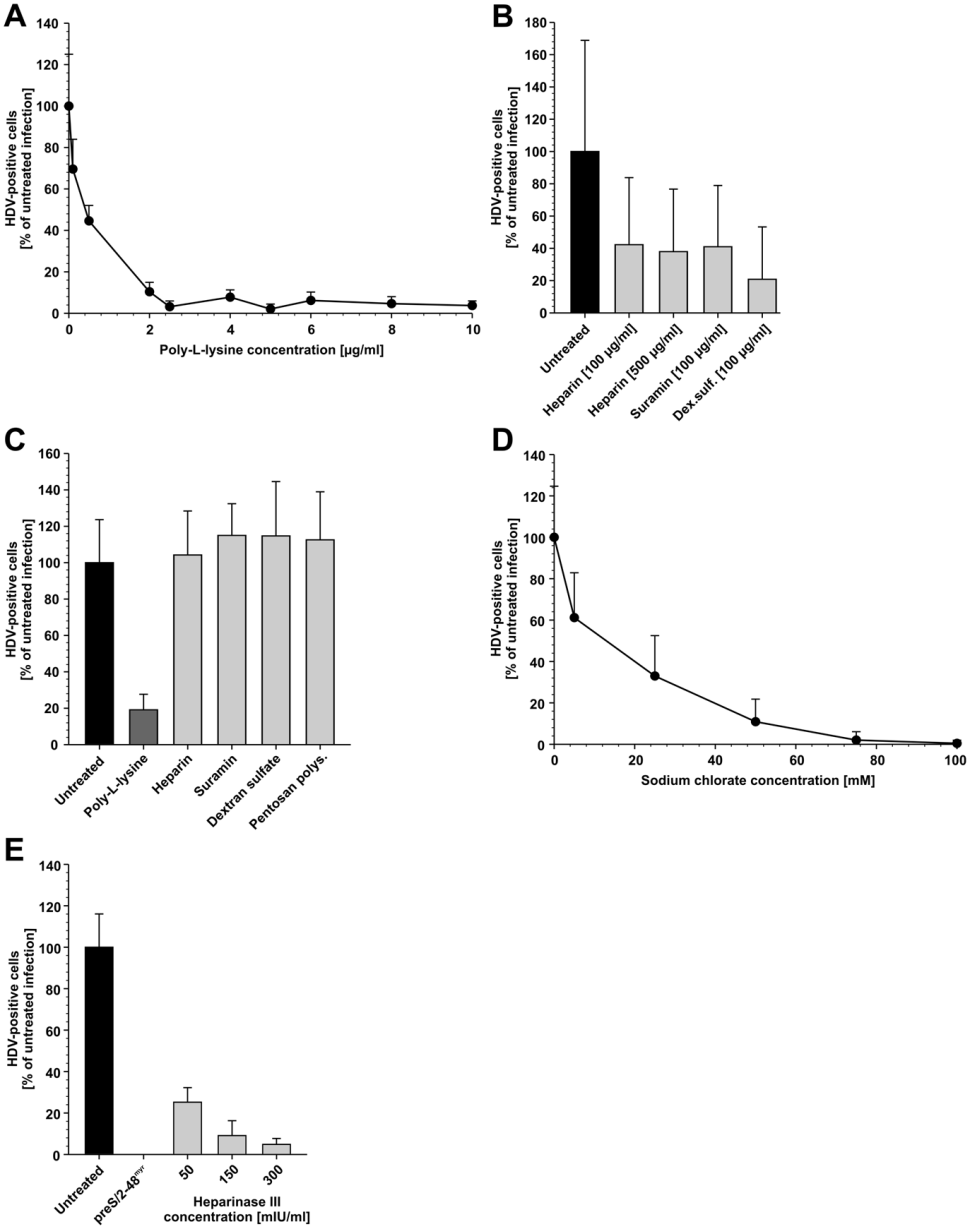
The obstruction or removal of negatively charged cellular interaction sites inhibit the HDV infection. **A**) HepaRG cells were pre-incubated with increasing concentrations of poly-L-lysine for 30 min at 37°C and subsequently infected in presence of the polycation for 16 h at 37°C. **B**) HDV was pre-incubated for 1 h at 37°C with heparin (100 µg/ml and 500 µg/ml), suramin (100 µg/ml) and dextran sulfate (100 µg/ml). Unbound polyanions were removed by ultrafiltration. HepaRG cells were incubated with the pre-treated viruses for 8 h at 37°C. **C**) HepaRG cells were pre-incubated with heparin, suramin, dextran sulfate, pentosan polysulfate or poly-L-lysine for 1 h at 37°C. Cells were washed and incubated with HDV for 8 h at 37°C in absence of the compounds. **D**) Sulfation of cellular proteoglycans was inhibited by treatment of HepaRG cells with increasing concentrations of sodium chlorate for 48 h prior to HDV infection. **E**) HepaRG cells were incubated for 1 h at 37°C with the indicated concentrations of heparinase III or 1 µM preS/2-48^myr^. The cells were washed and inoculated with HDV for 16 h at 37°C in presence of the substances. HDV-specific IF was performed for all experiments on day 6 p.i. Nuclei were stained with DAPI. The number of HDAg-positive cells and the total cell number were determined. The results are depicted as percentage of infected cells in comparison to the untreated control. In total, 822302 cells were counted for the analysis.

To substantiate the importance of cellular polyanionic binding sites for HDV, we blocked them by pre-incubating HepaRG cells with the polycation poly-L-lysine and infecting the cells in presence of the substance (figure 3A). Poly-L-lysine abolished HDV infection with an IC_50_ value of ∼0.5 µg/ml. To support the hypothesis, that poly-L-lysine blocks negatively charged binding sites on the cell, while polyanions address viral structures, two sets of experiments were performed. To test whether polyanions address viral structures, we pre-incubated HDV with heparin (100 and 500 µg/ml), suramin (100 µg/ml) or dextran sulfate (100 µg/ml) and removed unbound GAGs before infection of HepaRG. HDV infection was reduced to 21–42% in comparison to the untreated control (figure 3B). To demonstrate that poly-L-lysine interacts with cellular molecules, HepaRG cells were pre-incubated with heparin, suramin, dextran sulfate, pentosan polysulfate and poly-L-lysine at concentrations that inhibit HBV entry when present during virus inoculation (100 µg/ml, respectively 5 µg/ml for poly-L-lysine). Afterwards the cells were extensively washed and virus inoculation was performed in absence of the compounds. Pre-incubation of HepaRG cells with the polyanions did not impair infection, whereas pre-incubation with poly-L-lysine did (figure 3C). The inoculation time of HDV with HepaRG cells for the pre-incubation experiments shown in figure 3B and 3C was shortened to 8 h at 37°C in comparison to the other experiments of this study (16 h at 37°C). This change is based on the observation of our group, that shortening of the viral inoculation time during pre-incubation competition experiments increases the inhibitory effects of the compounds used (data not shown). To give additional evidence for the necessity of cellular proteoglycans for HDV infection, we inhibited the cellular adenosine triphosphate–sulfurylase and thereby sulfation of proteoglycans by addition of sodium chlorate to the culture medium [34]. A dose-dependent decrease of HDV-positive HepaRG cells (IC_50_ ≈ 13 mM) was observed upon sodium chlorate treatment, indicating the requirement of sulfated structures on the hepatocyte surface (figure 3D). To confirm, that GAG side chains of proteoglycans are responsible for HDV binding to hepatocytes we pre-incubated HepaRG cells with 50, 150 and 300 mIU/ml heparinase III and infected the cells with HDV (figure 3E). The selection of heparinase III for the experiment was based on our previous observation that this enzyme has a higher specific activity on the inhibition of HBV infection of HepaRG cells in comparison to heparinase I [20]. To avoid re-emergence of newly synthesized HSPG on the surface of HepaRG cells, virus inoculation was performed in presence of the enzyme for 16 h at 37°C. Heparinase III decreased the infection in a dose-dependent manner with a maximal reduction to 5% of the untreated control at 300 mIU/ml.

### P2XR do not Influence the in vitro HDV and HBV Infection

**Figure 4:**
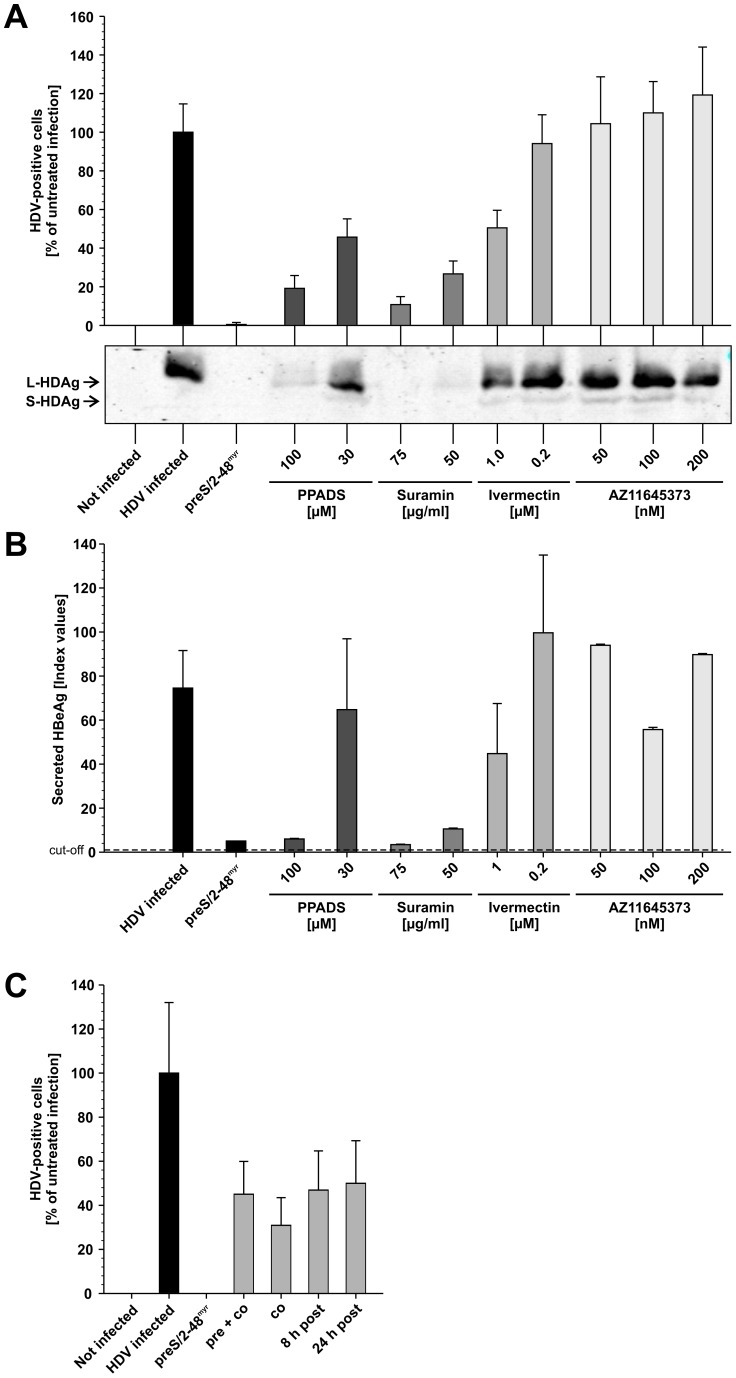
Inhibition of HDV and HBV infection by P2XR antagonists and agonists is dependent on their charge. A ) HepaRG cells were infected with serum of an HDV-positive patient (GTA7) in presence of P2XR antagonists (PPADS, suramin and AZ11645373) and agonists (ivermectin) for 16 h at 37°C. PreS/2-48^myr^ peptide (200 nM) was used in parallel as control. HDV-specific IF and nuclei staining was done 5 days p.i. The number of total and HDV-infected cells was quantified using ImageJ software. The results are depicted as percentage of the untreated control. In total, 290368 cells were counted for the analysis. Cell lysates of in parallel infected HepaRG cells were used for an HDAg-specific Western Blot. **B**) HepaRG cells were infected with HBV in presence of P2XR antagonists (PPADS, suramin and AZ11645373) and agonists (ivermectin) for 16 h at 37°C. Supernatants from days 8–11 p.i. were collected and secreted HBeAg was quantified by ELISA. Experiments were performed in duplicate and repeated at least two times independently. The values presented are mean ± SD. **C)** HepaRG cells were infected with serum of an HDV-positive patient (GTA7). Ivermectin (1 µM) was added at different time points during or after virus inoculation. HDV-specific IF and nuclei staining was done 6 days p.i. The number of total and HDV-infected cells was quantified using the ImageJ software. The results are depicted as percentage of the untreated control. In total, 128450 cells were counted for the analysis.

To analyze the contribution of P2XR in HBV/HDV entry, we transferred the infection experiments of Taylor *et al.* [25] to the HepaRG cell line and enclosed a set of P2XR antagonists and agonists originally not included. P2XR_4_ and P2XR_7_ expression in HepaRG cells was confirmed by PCR (data not shown), as the only data about P2XR expression in hepatic cells was available for the human hepatoma cell line Huh7 [29]. PHH or HepaRG cells were treated for 1 h with sulfated (PPADS, suramin) or non-sulfated (AZ11645373) P2XR antagonists as well as the non-sulfated P2XR agonist ivermectin and subsequently infected with HBV or HDV in presence of the compounds. Specificity of infection was controlled by preS/2-48^myr^. HDV infection was analyzed by HDV-specific IF and Western Blot on day 6 p.i. (figure 4A). Quantification of the IF showed that PPADS and suramin were able to inhibit the HDV infection by 81% (100 µM) and 89% (75 µg/ml), respectively. 1 µM ivermectin led to reduction of HDV infection in HepaRG cells by 49%. No influence on HDV infection was observed using lower ivermectin concentrations. AZ11645373 did not interfere with infection at concentrations up to 200 nM. The results on single cell level were supported by HDAg-specific Western blot analyses on HepaRG cell lysates (figure 4A, bottom). L- and S-HDAg specific bands were detected in the untreated but not in the preS/2-48^myr^ or mock treated control. HDV-specific signals were almost completely abrogated at 100 µM PPADS and 50 µg/ml suramin. Infection of HepaRG cells with HDV in presence of 1 µM ivermectin led to slight reduction of the HDAg-signals. No reduction of HDAg-signals was determined for AZ11645373 at concentrations up to 200 nM.

To investigate whether the effects observed with the purinergic antagonists and agonist in HDV infection also hold true for HBV, we performed a corresponding set of HBV infection experiments in HepaRG cells. HBV infection was quantified by measuring the secreted HBsAg and HBeAg between days 8–11 p.i. Figure 4B exemplarily depicts the HBeAg measurements. PPADS decreased the HBV infection by 13% at 30 µM and 91% at 100 µM. Suramin led to a reduction of infection by 86% at 50 µg/ml and 95% at 75 µg/ml. 1 µM ivermectin led to a reduction of HBeAg secretion by 40% while no effect was observed when 200 nM ivermectin was tested. AZ11645373 did not inhibit HBV infection at concentrations up to 200 nM.

To examine whether the inhibitory effect of high concentrations of ivermectin on HBV and HDV infection is due to interference with virus entry or replication, we added 1 µM ivermectin at different time points during or after virus inoculation (figure 4C). Addition of ivermectin, irrespectively if it was added during or after virus inoculation resulted in a reduction of HDV infection by around 60%. This indicates that ivermectin addresses a step in the HDV replication cycle independent of receptor interaction, excluding a role of P2XR as receptor. It also seems that ivermectin does not play a role in HBV release because addition of ivermectin to HepaRG cells 8 days after inoculation with HBV (when HBV infection is already established) had no effect on HBV antigen secretion (data not shown).

In summary, the results obtained for P2XR antagonists and agonist in HBV/HDV infection support the idea that interference with viral entry by these compounds is mediated by their charge and not because a direct involvement of P2XR.

## Discussion

HDV as a virusoid uses the envelope proteins of HBV for its assembly and infection. This makes it likely that HBV and HDV also share similar entry mechanisms, including receptor usage. It has been previously demonstrated that HBV entry is dependent on the initial interaction with HSPG as attachment receptors on the hepatocyte surface [19], [20]. Here, we provide evidence that HSPG also act as attachment receptors for HDV. This conclusion is based on the observations that HDV infection of HepaRG cells and PHH is sensitive to inhibition with negatively-charged compounds and enzymatic removal or obstruction of cellular binding sites (figures 2 and 3). Our data indicate that negative-charged GAGs and other highly sulfated compounds bind to positively charged structures on the HDV surface, while poly-L-lysine addresses cellular structures. HDV-HSPG interaction is thereby prevented, leading to abrogation of infection. Our conclusions, arguing for a role of HSPGs as attachment receptors for HDV are supported by a recent publication by Sureau *et al.*, demonstrating binding of HDV particles to immobilized heparin [35].

Interestingly, the results of the performed competition experiments using GAGs are comparable for HDV and HBV [20]. For example, the inhibitory activity of the compounds is in both cases dependent on the degree of sulfation. Higher sulfated GAGs or GAG variants are more effective than lower or non-charged substances. A possible explanation for that is the unique composition of liver heparan sulfate with strongly enriched sulfation at the distal ends of the GAG chains [23]. The IC_50_ values obtained in the infection inhibition experiments are similar but 1.3 to 2-fold lower for HDV in comparison to HBV which can be related to the different viral titers used for HBV and HDV infection experiments or to the different envelope protein L:S ratio between both viruses.

Suramin has broad antiparasitic and antiviral effects [36]. It interferes with the infection of parasites like plasmodium falciparum sporozoites [37], [38] or viruses like the human T-cell lymphotropic virus (HTLV-III)/lymphadenopathy-associated virus (LAV) [39], yellow fever virus [40] or herpes simplex viruses [41]. This activity however is mediated by different modes of action. For HIV it was demonstrated that suramin acts as a competitive inhibitor of the viral reverse transcriptase, inhibiting replication [42]. In contrast, suramin blocks HSV-1 infection by interfering with virus attachment to cell-associated HSPG [41]. For hepatitis viruses it was demonstrated, that suramin is able to inhibit the infection of primary woodchuck hepatocytes with HDV [33] and blocks the *in vitro* duck hepatitis B virus (DHBV) infection [33], HCV binding [43], [44] and HBV infection [20]. The initial studies indicated that suramin inhibits the DNA-polymerase of hepatitis viruses [45]. Subsequently, it was however shown that *in vivo* DHBV-replication can only be inhibited, when suramin is present during virus inoculation [32]. Furthermore, Funk *et al.* demonstrated that suramin reduces the binding of DNA-containing DHBV particles to primary duck hepatocytes [46]. Finally, we could show that suramin exerts its function on HBV by interfering with HSPG interaction on hepatocytes [20].

Based on these results and the observation that suramin also acts as an antagonist of purinergic receptors [25], Taylor *et al.* hypothesized that P2XR might be involved as receptor in HBV and HDV entry. They addressed this question by using a set of antagonists and analyzing their effect on HBV and HDV infection of PHH. However, their work presented several weak points. The antagonists chosen for the experiments (PPADS, brilliant blue G and suramin) contain a high negative charge, mediated by sulfonate and phosphate groups. Our results agree with those of Taylor *et al.* inasmuch as the two P2XR antagonists suramin and PPADS inhibited HBV and HDV infection of PHH (data not shown) and HepaRG cells (figure 4). As shown for suramin, the compounds are not selectively acting on P2XR, but also on other cellular molecules. Furthermore, PPADS, brilliant blue G and suramin are not highly specific for the P2XR isoforms present in hepatocyte membranes (isoforms 4 and 7). No experiments applying highly specific inhibitors of hepatocyte-specific P2XR were shown in the study of Taylor *et al.* They mentioned the usage of AZ11645373, a selective and potent inhibitor of human P2XR_7_ [47] that is the most abundant isoform in Huh7 cells [29]. However, in their hands this compound was highly toxic on PHH. In contrast to the report of Taylor *et al.*, treatment of HepaRG cells with concentrations (up to 200 nM) that have been shown to block P2XR_7_ activity (IC_50_ ≈ 90 nM) [47] were not toxic for HepaRG cells (figure 4) or PHH (data not shown). More importantly, AZ11645373 did not show any influence on HDV or HBV infection of HepaRG cells (figure 4), indicating that at least the hepatocyte-specific P2XR_7_ has no effect on viral entry. AZ11645373, in contrast to the other used P2XR inhibitors is non-charged, indicating an essential role of charge in the mechanism of inhibition. Moreover, when we used the non-charged P2XR_4_ (the other P2XR isoform present on Huh7 cells) agonist ivermectin in our HBV and HDV infection experiments, we found only a slight reduction of infection at 1 µM and no effect at 0.2 µM (figure 4A–B). As activation of P2XR by ivermectin is amongst others associated with increased cellular influx of calcium and sodium ions due to prolonged ion channel opening, formation of pores in the cell membrane or receptor tyrosine kinase activation, the observed effect on HDV and HBV infection is likely to be correlated with an unspecific side effect. This hypothesis is supported by the observation that addition of ivermectin during or after HDV inoculation (figure 4C) results in comparable reductions of infection, indicating that it does not interfere with receptor binding, but most likely with post-entry steps in the HDV life cycle.

Taken all of these results into account, we conclude that the observed effects on HDV and HBV entry by the P2XR antagonists are mediated by the negative charge of the different compounds, interfering with HDV-HSPG interaction and not by a direct role of P2XR. Furthermore, we showed that HDV infection of hepatocytes, similarly to HBV initiates with a low-affinity binding to cell-associated HSPG, suggesting that both viruses share similar entry mechanisms.
